# Multidirectional (auto- and hetero-) aggression in the practice of forensic psychiatry

**DOI:** 10.1192/j.eurpsy.2021.1898

**Published:** 2021-08-13

**Authors:** A. Kalashnikova

**Affiliations:** Laboratory Of Psychology, V. Serbsky National Medical Research Centre for Psychiatry and Narcology, Moscow, Russian Federation

**Keywords:** Aggression, risk assessment, motivational aggressiveness, combined autoagression and heteroagression

## Abstract

**Introduction:**

The problem of coexistence of heteroaggression and autoaggression most clearly manifests itself in the field of forensic psychiatry. For example, in Russia, about 25% of criminals who committed aggressive actions had a history of suicide attempts.

**Objectives:**

Identification of specific personality traits in individuals with multidirectional aggression.

**Methods:**

In a continuous one-step study, relatively sane adults of both sexes were examined: 38 persons undergoing forensic examination with multidirectional aggression and 34 violent criminals. A wide range of forensic psychological techniques is used to identify aggression, suicidogenic and inhibitors of aggression. Nonparametric statistical methods were used: Spearman rank correlation coefficient (r) and Mann-Whitney (U).

**Results:**

The leading role in the genesis of multidirectional aggression, in contrast to other types of aggression, playing the combination (p<0.01) to the presence of motivational aggressiveness (according to the Hand Test, U=556.6; p=0,046) and the willingness to show negative feelings at the slightest arousal (annoyance, irascibility according to BDHI, U=468.2; p=0,012), along with suicidal personal qualities, which is combined with the deficiency of auto- and heteroaggression inhibitors (value orientations; socio-normative, dispositional, communicative, emotional inhibitors, coping strategies, etc.). However, the psychological mechanisms of multidirectional aggression are relatively non-nosospecific and are similar in mentally healthy individuals and individuals with personality and organic mental disorders.

**Conclusions:**

Multidirectional aggression in view of the increased risk of recidivism and personal and public danger should be taken into account by forensic experts when recommending psychocorrective measures in places of deprivation of liberty.

**Disclosure:**

No significant relationships.

